# CRP–TyG index and risk of new‑onset central retinal artery occlusion and subsequent major adverse cardiovascular and cerebrovascular events: a propensity score‑matched cohort study

**DOI:** 10.1186/s40001-025-03807-6

**Published:** 2026-01-12

**Authors:** Hang Liu, Fangyuan Zhu, Hao Xie, Shuo Li, Faxi Wang, Liang Hu, Ting Chen, Xuan Xiao

**Affiliations:** 1 Department of Clinical Laboratory, Institute of Translational Medicine, Renmin Hospital of Wuhan University, No. 238 Jiefang Road, Wuhan, 430060 Hubei China; 2 Department of Ophthalmology, Renmin Hospital of Wuhan University, Wuhan, 430060 Hubei China

**Keywords:** Central retinal artery occlusion, C‑reactive protein–triglyceride‑glucose index, Risk and outcome, MACCEs

## Abstract

**Background:**

The C‑reactive protein–triglyceride glucose index (CTI), a composite marker of systemic inflammation and metabolic status, has been associated with cardiovascular and cerebrovascular risk. Its role in central retinal artery occlusion (CRAO) and subsequent adverse outcomes is unclear.

**Methods:**

In this cohort study, 122 CRAO patients and 488 matched controls who underwent coronary angiography without coronary artery disease were analyzed. CTI was calculated as 0.412 × ln [CRP (mg/L) + 0.5 × ln [TG × FPG(mg/dL)]. Logistic regression adjusted for demographic, clinical, laboratory, and medication variables was used to explore the relationship between CTI and the risk of CRAO. Restricted cubic spline models assessed the dose–response relationship between CTI and CRAO risk. The primary endpoint was CRAO and the secondary endpoint was major adverse cardiovascular and cerebrovascular events (MACCEs) during the 36‑month follow‑up.

**Results:**

Higher CTI was independently associated with CRAO (adjusted OR = 1.46, 95% CI 1.14–1.88; *P* = 0.003). Restricted cubic spline analysis identified a CTI threshold of 9.810. CRAO patients with CTI > 9.810 had a higher incidence of MACCEs (31.1% vs. 10.1%, *P* < 0.001), particularly acute coronary syndromes (20.3% vs. 7.1%, *P* < 0.001). In subgroup analysis, CRAO patients with CTI > 9.810 had the highest MACCEs rate (40.0%) and significantly greater risk of all‑cause death, stroke, and acute coronary syndromes (all adjusted *P* < 0.05) compared with other groups. Time‑to‑event analysis revealed that among all groups, CRAO patients with CTI > 9.810 had the shortest median time to MACCEs occurrence at 6 months (IQR,  4–11).

**Conclusions:**

Elevated CTI is independently associated with increased CRAO risk and predicts higher adverse MACCEs outcomes, suggesting CTI as a potential biomarker for risk stratification and secondary prevention in CRAO patients.

## Highlights/Importance

- Higher CTI was independently associated with an increased risk of CRAO, with restricted cubic spline analysis identifying a threshold of 9.810 for elevated risk. Patients with elevated CTI presented a higher incidence and earlier onset of MACCEs, particularly acute coronary syndromes, stroke, and all‑cause death. These findings suggest that CTI may serve as a practical biomarker for risk stratification and to guide early preventive strategies in CRAO patients, potentially reducing subsequent cardiovascular and cerebrovascular complications.

## Introduction

Central retinal artery occlusion (CRAO) is a vision-threatening ophthalmic emergency, often referred to as an “acute retinal stroke”, characterized by sudden embolic or thrombotic obstruction of the central retinal artery. This results in an abrupt cessation of retinal perfusion, precipitating ischemic injury to the inner retinal layers and frequently culminating in irreversible vision loss [1, 2]. Clinically, patients present with sudden, profound monocular visual impairment, often described metaphorically as a “curtain descending” over the affected eye, alongside the hallmark fundoscopic finding of a cherry-red spot. Beyond its ocular manifestations, CRAO is increasingly recognized as a marker of systemic vascular dysfunction, sharing pathophysiological mechanisms with atherosclerosis, coronary artery disease, and ischemic stroke. Epidemiological studies have demonstrated that patients with CRAO have a markedly increased risk of major adverse cardiovascular and cerebrovascular events (MACCEs), including myocardial infarction and stroke, underscoring the need for comprehensive systemic evaluation and prevention strategies [2–6].

Conventional serum biomarkers, including lipid profiles and single inflammatory markers, capture only isolated aspects of vascular pathology and may be insufficient for accurate risk stratification of CRAO [7–10]. The triglyceride-glucose (TyG) index, a surrogate marker for insulin resistance, has been reported to be elevated in individuals with CRAO and to serve as an independent predictor of its occurrence [11]. Recent research has shifted toward integrated biomarkers that reflect both inflammatory and metabolic dysregulation, two central drivers of atherosclerosis and its complications.

Expanding upon this concept, the C‑reactive protein–triglyceride‑glucose index (CTI) is a novel composite biomarker that combines systemic inflammation (CRP) with metabolic status (TyG index), which provides an integrated measure of vascular risk burden. Elevated CTI levels have been independently associated with increased incidence of coronary artery disease, ischemic stroke, and other MACCEs across diverse populations, frequently demonstrating superior prognostic performance compared with individual biomarkers [12–14].

Despite these observations, the relationship between CTI and CRAO, as well as its prognostic significance for subsequent MACCEs, remains unclear. This present study aimed to systematically investigate the association between CTI and CRAO risk, and to assess the prognostic value of CTI for MACCEs during long‑term follow‑up. We analyzed CRAO patients and propensity score‑matched controls without coronary artery disease (CAD) (1:4 ratio), matched for demographic and clinical covariates. Logistic regression assessed CTI–CRAO associations, and restricted cubic spline modeling explored dose–response relationships, identifying an optimal CTI threshold. Patients were stratified by this threshold to compare MACCEs incidence and time‑to‑event outcomes. This approach enables early identification of high‑risk CRAO individuals, guiding tailored prevention and management strategies that integrate ocular vascular findings with systemic cardiovascular risk assessment to improve visual prognosis, reduce adverse events, and enhance overall survival and quality.

## Materials and methods

### Study population and design

**Fig. 1:**
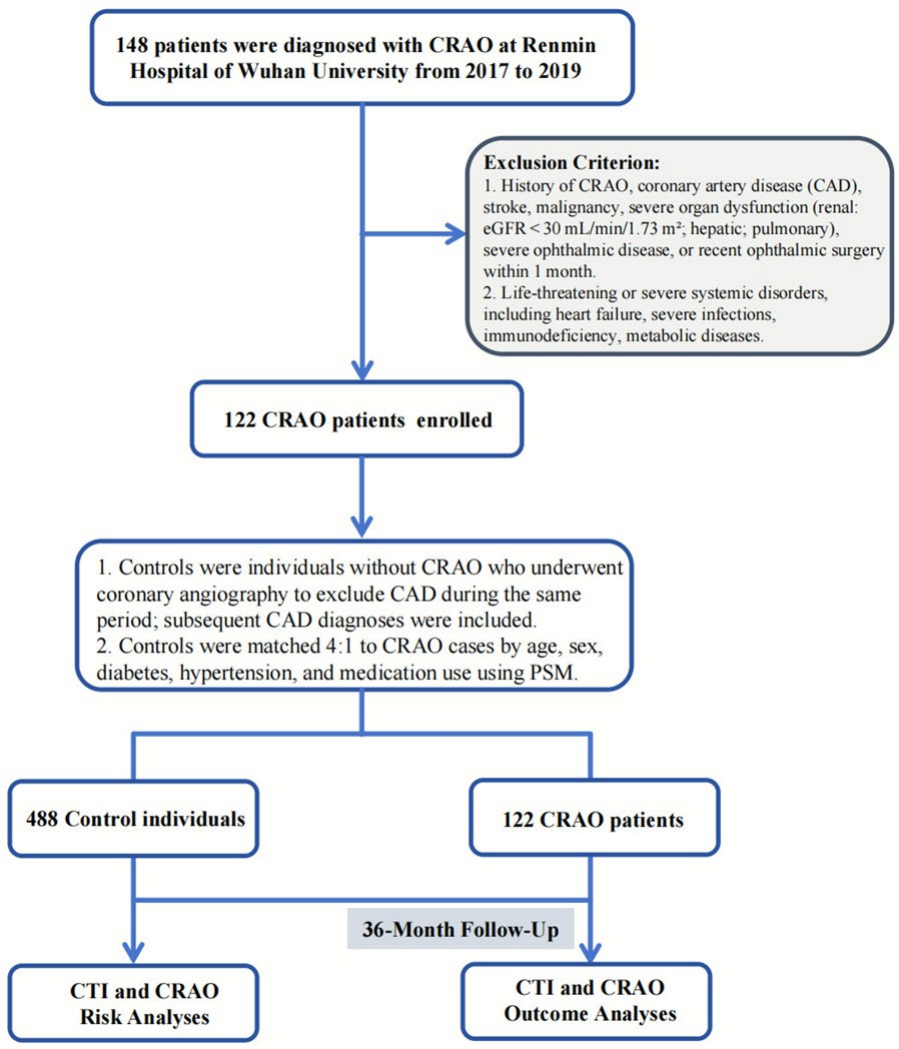
Flow diagram of patient selection. *CRAO* central retinal artery occlusion, *CAD* coronary artery disease, *PSM* propensity score matching, *CTI* C-reactive protein–triglyceride-glucose index

This study consisted of two components based on a retrospective analysis of electronic medical records from Renmin Hospital of Wuhan University between 2017 and 2019. The detailed inclusion and exclusion process is shown in Fig. 1. First, to evaluate the association between the CTI and CRAO, we conducted a retrospective case–control analysis. Patients diagnosed with CRAO were identified as cases, and individuals without CRAO who underwent coronary angiography during the same period formed the initial pool of potential controls. CRAO was diagnosed based on characteristic clinical and imaging findings. Funduscopic examination typically demonstrated diffuse retinal whitening of the posterior pole with a prominent cherry-red spot. Optical coherence tomography revealed inner retinal hyperreflectivity and thickening, consistent with acute ischemic injury. Fluorescein angiography showed delayed or absent filling of the central retinal artery. Differentiation from branch retinal artery occlusion was made by assessing the localized pattern of retinal whitening and arterial non-perfusion, and cases with cilioretinal artery sparing were identified by preserved perfusion in the cilioretinal territory. Two senior retinal specialists independently confirmed all diagnoses. Second, to assess the prognostic value of CTI for cardiovascular and cerebrovascular outcomes, a retrospective cohort follow-up was performed among all included participants. MACCEs were recorded through longitudinal follow-up.

CRAO patients did not routinely undergo coronary angiography because coronary imaging is not part of the standard diagnostic workflow for CRAO. Instead, all patients underwent comprehensive cardiovascular evaluation, including medical history, physical examination, ECG, and laboratory testing. Laboratory testing included routine cardiac biomarkers (troponin, CK-MB, and myoglobin) to assist in excluding acute cardiac injury or clinically evident CAD. Patients with any documented history or clinical evidence of CAD based on these evaluations were excluded. Additional exclusion criteria included: (1) prior CRAO, stroke, malignancy, severe organ dysfunction (renal: eGFR < 30 mL/min/1.73 m^2^; hepatic; pulmonary), severe ophthalmic disease, or recent ophthalmic surgery (within 1 month); and (2) life-threatening or severe systemic disorders, including heart failure, severe infections, immunodeficiency, and metabolic diseases (e.g., thyroid dysfunction, inherited metabolic disorders). Smoking status was categorized as current smoking versus non-smoking based on documented active tobacco use at hospitalization; drinking status was defined in the same manner.

Controls were selected from individuals without CRAO who underwent coronary angiography during the same period because of suspected coronary artery disease. Individuals with angiographically confirmed obstructive coronary disease were excluded, and only those with normal or non-obstructive coronary arteries were eligible as controls. The same exclusion criteria that applied to the CRAO group were used for the controls. All controls had complete clinical and laboratory data required for CTI calculation and covariate assessment. Subsequent cardiovascular events, including incident CAD during follow-up, were not exclusion criteria and were recorded as part of the MACCEs outcomes. Using propensity score matching (PSM), controls were matched 4:1 to CRAO cases based on age, sex, diabetes, hypertension, and medication use. This study was conducted in accordance with the Declaration of Helsinki and was approved by the Ethics Committee of Renmin Hospital of Wuhan University (Approval No. WDRY2022-K278). Written informed consent was obtained from all participants before enrollment.

### Clinical and laboratory data collection

Demographic characteristics, medical history, laboratory test results, and medication use were obtained from electronic medical records. The CTI was calculated as previously described [12], integrating systemic inflammation (CRP) and metabolic status (TyG) into a single composite biomarker:

$${\mathrm{CTI}} = 0.{\mathrm{412}} \times {\mathrm{ln}}\left[ {{\mathrm{CRP}}} \right] + 0.{\mathrm{5}} \times {\mathrm{ln}}\left[ {{\mathrm{TG}} \times {\mathrm{FPG}}} \right],$$

where CRP is expressed in mg/L, and triglycerides (TG) and fasting plasma glucose (FPG) are expressed in mg/dL.

### Outcome definitions and follow‑up

Because the association between CTI and CRAO was assessed using a retrospective case–control design, CRAO status itself was not treated as a study outcome but rather as the condition used to define case and control groups. The follow-up outcomes were evaluated in the second component of the study, which was conducted as a retrospective cohort among all included participants.

The primary follow-up outcome was the occurrence of MACCEs during 36 months of follow-up. MACCEs were defined as a composite endpoint that included cardiovascular death, non-fatal myocardial infarction, non-fatal ischemic or hemorrhagic stroke, hospitalization for heart failure, and any coronary or cerebrovascular revascularization procedure. Follow-up data were obtained from electronic medical records and the institutional follow-up system.

### Statistical analysis

Missing data were minimal (< 2% for all variables). A complete-case analysis was performed, and records with missing values were excluded from the relevant analyses. No data imputation was conducted. Continuous variables were expressed as mean ± standard deviation or median (interquartile range), and categorical variables as frequencies (percentages). Between-group comparisons were performed using the Student’s *t*-test or Mann–Whitney *U* test for two-group comparisons of continuous variables, and the *χ*^2^ test or Fisher’s exact test for categorical variables. For comparisons involving more than two groups, ANOVA or Kruskal–Wallis rank-sum test was used for continuous variables, and the *χ*^2^ test for trend or Kruskal–Wallis test for categorical variables, as appropriate. Post hoc pairwise comparisons were adjusted for multiple testing using the Bonferroni correction method. Multivariate logistic regression models were applied to evaluate the association between CTI and CRAO risk, adjusting for potential confounders. To explore potential non-linear relationships between CTI and outcomes, restricted cubic spline (RCS) analyses were performed using the rcssci package in R. A *P*-value < 0.05 was considered statistically significant. All analyses were conducted using R software (version 4.5.1) and SPSS (version 22.0).

## Results

### Characteristics of study participants

A total of 610 participants were included, comprising 122 CRAO cases and 488 matched controls. Baseline characteristics are summarized in Table 1. Compared with controls, CRAO patients had significantly higher neutrophil counts (3.94 ± 1.49 vs. 3.62 ± 1.24 × 10⁹/L, *P* = 0.011), neutrophil-to-lymphocyte ratio (NLR) (2.19 vs. 1.92, *P* = 0.005), total cholesterol (TC)(4.77 ± 1.06 vs. 4.39 ± 1.04 mmol/L, *P* < 0.001), triglycerides (TG) (2.12 ± 1.68 vs. 1.66 ± 0.98 mmol/L, *P* < 0.001), LDL-cholesterol (2.74 ± 0.86 vs. 2.39 ± 0.79 mmol/L, *P* < 0.001), and hs-CRP (1.28 vs. 0.81 mg/L, *P* = 0.016). Both TyG index and CTI were significantly higher in the CRAO group (TyG: 8.90 ± 0.57 vs. 8.72 ± 0.54, *P* = 0.001; CTI: 9.01 ± 0.95 vs. 8.64 ± 0.89, *P* < 0.001).

**Table 1:** Baseline characteristics of study participants

Characteristics	Total *n* = 610	Control group *n* = 488	CRAO group *n* = 122	*P* value
Clinical variables				
Male sex (%)	324 (53.1)	258 (52.9)	66 (54.1)	0.808
Age (years)	62 (55, 68)	62 (58, 68)	63 (55, 70)	0.510
Hypertension (%)	271 (44.4)	217 (44.5)	54 (44.3)	0.968
Diabetes (%)	77 (12.6)	56 (11.5)	21 (17.2)	0.088
Current smoking status (%)	132 (21.7)	108 (21.1)	24 (19.8)	0.583
Current drinking status (%)	68 (11.1)	58 (11.9)	10 (8.2)	0.247
Fatty liver (%)	16 (2.6)	11 (2.3)	5 (4.1)	0.254
Laboratory variables				
WBC (10^9^/L)	6.03 ± 1.62	6.05 ± 1.56	6.13 ± 1.83	0.446
Neu (10^9^/L)	3.68 ± 1.30	3.62 ± 1.24	3.94 ± 1.49	**0.011**
Lym (10^9^/L)	1.77 ± 0.58	1.79 ± 0.57	1.69 ± 0.61	0.120
NLR	2.02 (1.53, 2.63)	1.92 (1.49, 2.57)	2.19 (1.64, 2.95)	**0.005**
Tch (mmol/L)	4.47 ± 1.05	4.39 ± 1.04	4.77 ± 1.06	** < 0.001**
TG (mmol/L)	1.76 ± 1.17	1.66 ± 0.98	2.12 ± 1.68	** < 0.001**
HDL-ch (mmol/L)	1.16 ± 0.30	1.17 ± 0.30	1.13 ± 0.31	0.188
LDL-ch (mmol/L)	2.46 ± 0.82	2.39 ± 0.79	2.74 ± 0.86	** < 0.001**
Glu (mmol/L)	5.34 ± 1.31	5.36 ± 1.31	5.24 ± 1.28	0.919
Cr (μmol/L)	66 (55, 78)	66 (54, 77)	67 (56, 85)	0.051
eGFR (ml/min/1.73m^2^)	95 (87, 103)	95 (87, 102)	95 (83,105)	0.684
hs-CRP (mg/L)	0.88 (0.27, 2.46)	0.81 (0.26, 2.26)	1.28 (0.33, 3.85)	**0.016**
TYG	8.76 ± 0.55	8.72 ± 0.54	8.90 ± 0.57	**0.001**
CTI	8.71 ± 0.92	8.64 ± 0.89	9.01 ± 0.95	** < 0.001**
Medications variables				
Aspirin (%)	383 (62.8)	312 (63.9)	71 (58.2)	0.241
Statins (%)	452 (74.1)	365 (74.8)	87 (71.3)	0.432
β-Blocker (%)	140 (23.0)	112 (23.0)	28 (23.0)	1.000
ACEI/ARB (%)	66 (10.8)	51 (10.5)	15 (12.3)	0.558
CCB (%)	129 (21.1)	103 (21.1)	26 (21.3)	0.960

*WBC* white blood cell, *Neu* neutrophil, *Lym* lymphocyte, *NLR* neutrophil-to-lymphocyte ratio, *Tch* total cholesterol, *TG* triglycerides, *HDL-ch* high-density lipoprotein cholesterol, *LDL-ch* low-density lipoprotein cholesterol, *Glu* glucose, *CR* Creatinine, *eGFR* estimated glomerular filtration rate, *hs-CRP* hypersensitive C-reactive protein, *TYG* (triglyceride-glucose index) is calculated using the formula: TYG index = ln[(fasting triglycerides (mg/dL) × Fasting glucose (mg/dL)/2], *CTI* (C-reactive protein–triglyceride glucose index) is calculated through the formula: 0.412 × Ln [(high-sensitivity C-reactive protein(mg/L)] + ln[(fasting triglycerides (mg/dL) × fasting glucose (mg/dL)/2]. *ACEI/arb* angiotensin-converting enzyme inhibitor/angiotensin Ⅱ receptor blocker, *CCB* calcium channel blocker

### Association between CTI and CRAO risk

In unadjusted logistic regression, each unit increase in CTI was associated with an 80.8% higher odds of CRAO (OR = 1.808, 95% CI 1.269–2.574, *P* = 0.001) (Table 2). This association remained robust after sequential adjustments for demographic factors, comorbidities, laboratory indicators, and medication use (Model 2: OR = 1.464, 95% CI 1.138–1.882, *P* = 0.003). In quartile analysis, participants in the highest CTI quartile (≥ 9.32) had over twice the odds of CRAO compared to the lowest quartile (Q1) (Model 2 OR = 2.356, 95% CI 1.199–4.626, *P* = 0.013; *P* for trend = 0.023).

**Table 2:** Logistic regression analyses of the association between CTI and CRAO

Variables		Crude		Model 1		Model 2	
		OR (95%CI)	*P*-value	OR (95%CI)	*P*-value	OR (95%CI)	*P*-value
CTI		1.808 (1.269–2.574)	0.001	1.839 (1.281, 2.640)	0.001	1.464 (1.138, 1.882)	0.003
CTI (category)	Range						
Q1	≤ 8.05	1(ref)		1(ref)		1(ref)	
Q2	8.05–8.73	1.779 (0.955, 3.312)	0.069	1.881 (0.988, 3.579)	0.054	1.740 (0.880, 3.438)	0.111
Q3	8.73–9.32	1.707 (0.914, 3.189)	0.093	1.865 (0.984, 3.536)	0.056	1.625 (0.822, 3.212)	0.163
Q4	≥ 9.32	2.673 (1.470, 4.860)	0.001	2.811 (1.515, 5.217)	0.001	2.356 (1.199, 4.626)	0.013
*P* for trend			0.002		0.002		0.023

Crude: no adjustment

Model 1: adjusted for sex, age, hypertension, diabetes, fatty liver, smoking status, and drinking status

Model 2: adjusted for all variables in Model 1, plus the following laboratory indicators and medication use: laboratory indicators: white blood cell count, neutrophils, lymphocytes, neutrophil-to-lymphocyte ratio, triglycerides, high-density lipoprotein cholesterol, low-density lipoprotein cholesterol, total cholesterol/high-density lipoprotein cholesterol ratio, creatinine, estimated glomerular filtration rate, and glucose. Medication use: aspirin, statins, β-blockers, angiotensin-converting enzyme inhibitors/angiotensin Ⅱ receptor blockers, and calcium channel blockers

**Fig. 2:**
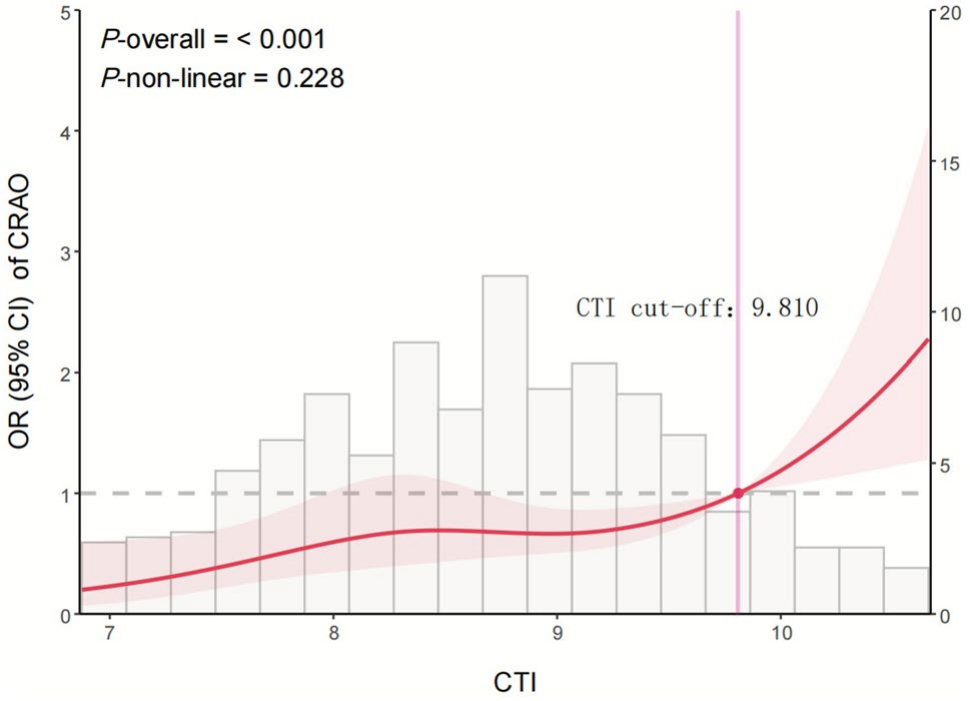
Restricted cubic spline curve showing the association between CTI and the risk of CRAO. The solid red line represents the odds ratio (OR), and the shaded area indicates the 95% confidence interval

RCS analysis confirmed a significant overall association between CTI and CRAO risk (*P*-overall < 0.001), with no evidence of non-linearity (*P* for non-linearity = 0.228) (Fig. 2). CRAO risk increased steadily with CTI, with a steeper rise observed above approximately 9.810.

### Subgroup analyses

The positive association between CTI and CRAO was largely consistent across subgroups (Table 3), with no significant effect modification by age, sex, hypertension, diabetes, smoking status, lipid levels, or most medication categories (*P* for interaction > 0.05), except for statin use (*P* for interaction = 0.019), where the association was stronger in non-users (OR = 3.011, 95% CI 1.536–5.903, *P* = 0.001).

**Table 3:** Subgroup analysis of the association between CTI and CRAO in model 2

Subgroup	CRAO		
	OR (95% CI)	*P* value	*P* for interaction
Age			0.255
< 60	1.595 (1.003, 2.537)	0.049	
≥ 60	1.861 (1.291, 2.681)	0.001	
Sex			0.671
Male	1.536 (1.058, 2.229)	0.024	
Female	1.797 (1.147, 2.816)	0.011	
Hypertension			0.932
No	2.224 (1.279, 3.867)	0.005	
Yes	1.638 (0.864, 3.104)	0.131	
Diabetes			0.373
No	1.860 (1.227, 2.818)	0.003	
Yes	1.342 (0.211, 8.554)	0.755	
Smoking status			0.859
No	1.683 (1.131, 2.504)	0.010	
Yes	0.519 (0.041, 6.560)	0.612	
WBC			0.300
≤ 5.81	1.337 (0.902, 1.981)	0.148	
> 5.81	1.764 (1.159, 2.685)	0.008	
NEU			0.674
≤ 3.41	1.638 (1.077, 2.492)	0.021	
> 3.41	1.667 (1.119, 2.483)	0.012	
LYM			0.953
≤ 1.70	1.653 (1.151, 2.374)	0.007	
> 1.70	1.716 (1.082, 2.721)	0.022	
NLR			0.703
≤ 2.02	1.806 (1.144, 2.851)	0.011	
> 2.02	1.622 (1.128, 2.332)	0.009	
CR			0.681
≤ 66	1.597 (1.115, 2.287)	0.011	
> 66	2.060 (1.312, 3.235)	0.002	
eGFR			0.488
≤ 95	1.989 (1.268, 3.122)	0.003	
> 95	1.374 (0.962,1.964)	0.081	
TC			0.919
≤ 4.36	1.687 (1.023, 2.781)	0.040	
> 4.46	1.494 (1.052, 2.122)	0.025	
HDL			0.237
≤ 1.11	1.307 (0.864, 1.979)	0.205	
> 1.11	1.950 (1.273, 2.987)	0.002	
LDL			0.468
≤ 2.41	2.456 (1.544, 3.939)	< 0.001	
> 2.41	1.240 (0.859, 1.790)	0.250	
Aspirin			0.558
No	2.070 (1.201, 3.567)	0.009	
Yes	1.798 (1.241, 2.606)	0.002	
Statins			**0.019**
No	3.011 (1.536, 5.903)	0.001	
Yes	1.329 (0.940, 1.877)	0.107	
β-Blocker			0.817
No	1.602 (1.171, 2.190)	0.003	
Yes	1.780 (0.829, 3.822)	0.139	
ACEI/ARB			0.970
No	1.582 (1.195, 2.092)	0.001	
Yes	–	0.999	
CCB			0.682
No	1.761 (1.299, 2.387)	< 0.001	
Yes	1.941 (0.717, 5.259)	0.192	

*WBC* white blood cell, *Neu* neutrophil, *Lym* lymphocyte, *NLR* neutrophil-to-lymphocyte ratio, *Tch* total cholesterol, *TG* triglycerides, *HDL-ch* high-density lipoprotein cholesterol, *LDL-ch* low-density lipoprotein cholesterol, *Glu* glucose, *CR* Creatinine, *eGFR* estimated glomerular filtration rate, *hs-CRP* hypersensitive C-reactive protein, *TYG* (triglyceride-glucose index) is calculated using the formula: TYG index = ln[(fasting triglycerides (mg/dL) × fasting glucose (mg/dL)/2], *CTI* (C-reactive protein–triglyceride glucose index) is calculated through the formula: 0.412 × Ln [(high-sensitivity C-reactive protein(mg/L)] + ln[(fasting triglycerides (mg/dL) × fasting glucose (mg/dL)/2]. *ACEI/ARB* angiotensin-converting enzyme inhibitor/angiotensin Ⅱ receptor blocker, *CCB* calcium channel blocker

Model 2: adjusted for all variables in Model 1, plus the following laboratory indicators and medication use: laboratory indicators: white blood cell count, neutrophils, lymphocytes, triglycerides, high-density lipoprotein cholesterol, low-density lipoprotein cholesterol, total cholesterol/high-density lipoprotein cholesterol ratio, creatinine, estimated glomerular filtration rate, and glucose. Medication use: aspirin, statins, β-blockers, angiotensin-converting enzyme inhibitors/angiotensin Ⅱ receptor blockers, and calcium channel blockers

### Follow‑up outcomes

During a median follow‑up of 13 months (IQR, 8–20 months), participants with CTI > 9.810 had a significantly higher incidence of MACCEs compared with those with CTI ≤ 9.810 (31.1% vs. 10.1%, *P* < 0.001). Notably, the rate of acute coronary syndromes was markedly elevated in the high‑CTI group (20.3% vs. 7.1%, *P* < 0.001). Although the incidences of stroke (8.1% vs. 3.4%, *P* = 0.059) and atrial fibrillation (5.4% vs. 1.9%, *P* = 0.078) were higher in the high‑CTI group, these differences did not reach statistical significance. No significant associations were found between CTI level and all‑cause death, cardiac death, revascularization, or heart failure (Table 4).

**Table 4:** Association between CRP–TyG index (CTI) level and follow-up clinical outcomes in all participants

Follow-up clinical outcomes	Total (*n* = 610)	CTI ≤ 9.810 (*n* = 536)	CTI > 9.810 (*n* = 77)	*P* value
MACCEs (%)	77 (12.6)	54 (10.1)	23 (31.1)	** < 0.001**
All-cause death (%)	3 (0.5)	2 (0.4)	1 (1.4)	0.322
Cardiac death (%)	2 (0.3)	1 (0.2)	1 (1.4)	0.228
Revascularization (%)	7 (1.1)	5 (0.9)	2 (2.7)	0.204
Stroke (%)	24 (3.9)	18 (3.4)	6 (8.1)	0.059
Acute coronary syndromes (%)	52 (8.7)	38 (7.1)	15 (20.3)	** < 0.001**
Atrial fibrillation (%)	14 (2.3)	10 (1.9)	4 (5.4)	0.078
Heart failure (%)	4 (0.7)	4 (0.7)	0 (0.0)	1.000
Follow-up duration until MACCEs (months, IQR)	13 (8, 20)	14 (8, 20)	12 (8, 21)	0.802

*MACCEs* major adverse cardiovascular and cerebrovascular events

**Fig. 3:**
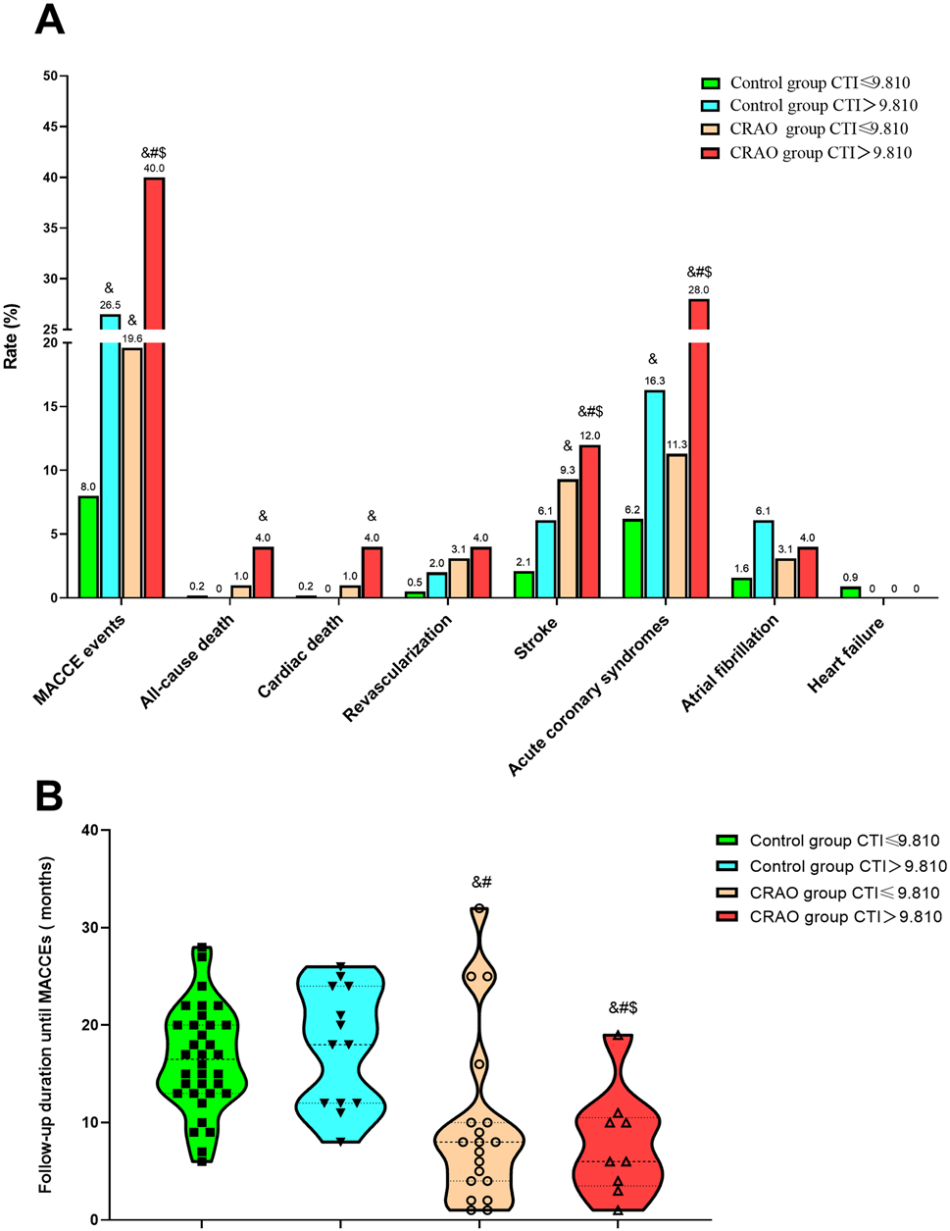
Association between CTI levels and follow‑up clinical outcomes.** A** Incidence of major adverse cardiovascular and cerebrovascular events (MACCEs);** B** follow‑up duration until MACCEs occurrence. &, compared with the control group, CTI ≤ 9.810, *P* < 0.05; #, compared with the control group, CTI > 9.810, *P* < 0.05; $, compared with the CRAO group, CTI ≤ 9.810, *P* < 0.05

To further elucidate the joint impact of CRAO status and CTI level on adverse vascular outcomes, participants were stratified into four subgroups: control group with CTI ≤ 9.810, control group with CTI > 9.810, CRAO group with CTI ≤ 9.810, and CRAO group with CTI > 9.810. Marked differences in MACCEs incidence were observed across these strata (Fig. 3A). The CRAO group with CTI > 9.810 exhibited the highest MACCE rate (40.0%) compared with the control group with CTI ≤ 9.810 (8.0%), the control group with CTI > 9.810 (26.5%), and the CRAO group with CTI ≤ 9.810 (19.6%) (all adjusted *P* < 0.05). Moreover, the CRAO group with CTI ≤ 9.810 also demonstrated a significantly higher MACCEs incidence than the control group with CTI ≤ 9.810 (adjusted *P* < 0.05).

For specific outcomes, the CRAO group with CTI > 9.810 had significantly higher rates of all‑cause death (4.0% vs. 0.2%, adjusted *P* < 0.05 vs. control group with CTI ≤ 9.810), stroke (12.0% vs. 9.3%, 6.1%, and 2.1% in the other three groups, all adjusted *P* < 0.05), and acute coronary syndromes (all adjusted *P* < 0.05 vs. other three groups). Time‑to‑event analysis (Fig. 3B) showed that the CRAO group with CTI > 9.810 had the shortest median time to MACCEs occurrence (6 months [IQR, 4–11]), followed by the CRAO group with CTI ≤ 9.810 (8 months [IQR, 5–21]), the control group with CTI ≤ 9.810 (12 months [IQR, 8–22]), and the control group with CTI > 9.810 (17 months [IQR, 13–20]).

## Discussion

In this cohort study, we found that the CTI, an integrated measure of systemic inflammation and metabolic dysfunction, was independently associated with both the presence of CRAO and the subsequent occurrence of MACCEs during a 36-month follow-up period. CTI levels were significantly higher in patients with CRAO compared with PSM matched controls, and this association persisted after rigorous adjustment for demographic, clinical, laboratory, and medication variables. RCS modeling revealed an inflection point at approximately 9.810, above which the risks of CRAO and MACCEs rose markedly. These results suggest that CTI may serve as a valuable biomarker for simultaneous stratification of ocular and systemic vascular risk, bridging the gap between ophthalmic findings and broader cardiovascular prevention.

CRAO is a vision-threatening ophthalmic emergency and an important indicator of systemic vascular disease [1, 4]. It shares common risk factors with systemic vascular conditions, including hypertension, diabetes, and lipid metabolism disorders, which contribute to atherosclerosis and thromboembolism [12, 13]. Lipid abnormalities, particularly elevated LDL-C and non-HDL-C, are prevalent in CRAO patients and may promote carotid plaque formation and rupture, a common source of retinal emboli [7, 8, 15, 16]. Previous studies have identified hs-CRP as an independent predictor of CRAO [7, 10]. As a liver-derived acute-phase protein primarily induced by interleukin-6, hs-CRP not only serves as a marker of systemic inflammation but also contributes directly to endothelial dysfunction, carotid atherogenesis, plaque instability, and thrombosis [17, 18]. These roles highlight its pivotal involvement in the pathophysiology of CRAO, ischemic stroke, and transient ischemic attack.

Emerging evidence emphasizes that vascular injury results from the interplay between lipid dysregulation and chronic inflammation, which synergistically drive the pathogenesis of atherosclerosis, plaque rupture, and thromboembolic events [19–21].

The CRP–TyG index, by combining markers of inflammation (CRP) and insulin resistance (TyG index), offers a comprehensive tool for vascular risk assessment. The TyG index alone has been identified as an independent predictor of CRAO (HR = 1.84, 95% CI = 1.19–4.23), emphasizing the critical role of metabolic dysfunction in its pathogenesis [11]. Recent large-scale cohort studies have demonstrated that the CTI is a robust predictor of cardiovascular and cerebrovascular disease risk across diverse metabolic profiles. Large-scale cohort studies, including data from CHARLS and NHANES, have consistently demonstrated that the CTI robustly predicts cardiovascular and cerebrovascular disease risk across diverse metabolic profiles. Elevated CTI is strongly associated with increased risk of total CVD, congestive heart failure, coronary heart disease, myocardial infarction, and stroke, with consistent effects across sex and age groups [14, 22]. Moreover, both cumulative exposure to high CTI and dynamic increases over time independently correlate with heightened cardiovascular risk, especially among middle-aged and elderly individuals [23]. Notably, the association between CTI and stroke risk is particularly pronounced in individuals with normal glucose regulation and prediabetes [13]. These findings align closely with our results, which demonstrate a significant association between elevated CTI and increased risk of CRAO. Our study further emphasizes the potential of CTI to identify high-risk individuals who may benefit from targeted preventive interventions, underscoring the importance of integrating CTI into clinical risk assessment for earlier detection and management of vascular risk.

In this study, elevated CTI was associated not only with an increased risk of CRAO, but also with a higher incidence and earlier onset of MACCEs, including myocardial infarction, stroke, and cardiovascular death, during long-term follow-up. Time-to-event analysis highlighted that patients with higher CTI experienced MACCEs significantly sooner than those with lower CTI, emphasizing its prognostic value. Consistent with these findings, recent large-scale studies have demonstrated that elevated CTI robustly predicts adverse cardiovascular outcomes and mortality. For instance, NHANES data revealed that higher CTI levels significantly increase risks of cardiovascular mortality (HR 2.28; 95% CI 1.69–3.24) and all-cause mortality (HR 2.14; 95% CI 1.76–2.55) [22]. Longitudinal cohort analyses have further established a dose–response relationship between cumulative CTI exposure and incident cardiovascular disease, with sustained high CTI trajectories linked to worse clinical outcomes [23]. Moreover, the CHARLS study confirmed that combined elevations in TyG and hsCRP synergistically elevate risks of coronary heart disease and stroke, partially mediating cardiovascular mortality [13, 24]. Together, these results support CTI as a comprehensive biomarker reflecting underlying pathophysiology and a powerful predictor of long-term cardiovascular, cerebrovascular, and mortality outcomes across diverse populations.

The CRP–TyG index captures the synergistic effects of chronic inflammation and insulin resistance, which converge on several molecular pathways that drive endothelial dysfunction, plaque vulnerability, and thrombosis, which together could explain the observed associations between elevated CTI, CRAO, and subsequent MACCEs. CRP promotes endothelial injury, vascular smooth muscle proliferation, and destabilization of atherosclerotic plaques [19, 25]. Simultaneously, the TyG index reflects insulin resistance, which exacerbates atherosclerosis by stimulating the production of sdLDL-C, activating adipose tissue inflammation, and elevating circulating free fatty acids [26, 27]. When both CRP and TyG are elevated, they may jointly activate pro-inflammatory and pro-thrombotic signaling pathways, including NF-κB, JNK, and TLR4, resulting in endothelial activation, local immune cell infiltration, and a hypercoagulable state [28, 29]. On the other hand, high CRP impairs endothelial nitric oxide synthesis, while TyG-related insulin resistance inhibits the PI3K–Akt–eNOS pathway, collectively diminishing endothelial vasoprotective function and promoting vasoconstriction [30–32]. Given that CRAO represents an occlusion of terminal retinal arteries, it may be a localized manifestation of broader systemic microvascular dysfunction. Elevated CTI could therefore reflect widespread vascular injury. Additionally, prior studies have shown that CRP enhances tissue factor expression and coagulation activation, and promotes platelet aggregation via the CD40-CD40L axis [33–35]. Insulin resistance and hypertriglyceridemia further aggravate blood viscosity and thrombotic risk, potentially accelerating the occurrence of macrovascular events. Given the robust epidemiological associations observed in our study, future research should explore the underlying biological mechanisms linking CTI to vascular outcomes.

Despite offering important insights, this study has limitations. First, its retrospective, single‑center design may introduce selection bias and residual confounding, although PSM was applied to enhance internal validity. Second, as a novel biomarker, the CTI’s optimal threshold and clinical utility require confirmation in larger prospective cohorts; the relatively high CTI values in our study may partly reflect the selection of controls without CAD. Third, the generalizability of these findings to other vascular‑risk populations remains uncertain, and the mechanistic pathways linking CTI to vascular events remain speculative. Future multicenter, long‑term studies should validate the predictive value of CTI, assess its integration with imaging or emerging biomarkers (e.g., FGF21), and test whether interventions targeting inflammation or insulin resistance can mitigate MACCEs risk in CRAO [36, 37]. Nevertheless, these results support CTI as a promising tool for vascular risk stratification and a potential target for personalized prevention.

## Conclusions

CTI is independently associated with the risk of CRAO and predicts subsequent MACCEs over 36 months, with a threshold of approximately 9.810 marking substantially higher risk. These findings suggest that CTI could serve as a valuable biomarker for integrated ocular–cardiovascular risk assessment, supporting the need for proactive systemic evaluation and preventive care in patients with elevated CTI, regardless of CRAO status.

## Data Availability

The datasets generated during and analyzed during the current study are not publicly available due to privacy or ethical restrictions but are available from the corresponding author on reasonable request.

## Abbreviations

CAD: Coronary artery disease
CI: Confidence interval
CRAO: Central retinal artery occlusion
CRP: C-reactive protein
CTI: C-reactive protein-triglyceride glucose index
FBG: Fasting plasma glucose
HDL-ch: High-density lipoprotein cholesterol
hs-CRP: High-sensitivity C-reactive protein
IQR: Interquartile range
LDL-ch: Low-density lipoprotein cholesterol
Lym: Lymphocyte
MACCEs: Major adverse cardiovascular and cerebrovascular events
Neu: Neutrophil
NLR: Neutrophil-to-lymphocyte ratio
OR: Odds ratio
PSM: Propensity score matching
RCS: Restricted cubic spline
Tch: Total cholesterol
TG: Triglyceride
TYG: Triglyceride-glucose index
WBC: White blood cell
